# Exploring Chitosan Lactate as a Multifunctional Additive: Enhancing Quality and Extending Shelf Life of Whole Wheat Bread

**DOI:** 10.3390/foods13101590

**Published:** 2024-05-20

**Authors:** Pratik Singh, Vikas Yadav, Deblu Sahu, Krishan Kumar, Doman Kim, Deng Yang, Sivaraman Jayaraman, Maciej Jarzębski, Marek Wieruszewski, Kunal Pal

**Affiliations:** 1 Department of Life Sciences, Parul Institute of Applied Science, Parul University, Vadodara 391760, Gujarat, India; pratiksingh020801@gmail.com (P.S.); krishan.kumar20140@paruluniversity.ac.in (K.K.); 2 Department of Biotechnology & Medical Engineering, National Institute of Technology Rourkela, Rourkela 769008, Odisha, India; 3 Department of International Agricultural Technology & Institute of Green Bioscience and Technology, Seoul National University, Seoul 151-742, Republic of Korea; 4 College of Food Science and Engineering, Qingdao Agriculture University, No. 700 Chancheng Road, Qingdao 266109, China; 5 Department of Physics and Biophysics, Faculty of Food Science and Nutrition, Poznan University of Life Sciences, 60-637 Poznan, Poland; 6 Department of Mechanical Wood Technology, Faculty of Forestry and Wood Technology, Poznan University of Life Sciences, 60-627 Poznan, Poland

**Keywords:** bakery, chitosan derivatives, overall quality, polysaccharides, storage life, texture

## Abstract

The shelf life of whole wheat bread (WWB) significantly impacts its freshness and overall quality. This research investigated the impact of chitosan lactate (CL) on various characteristics influencing the shelf life of WWB, including its physical, chemical, textural, antimicrobial, and sensory attributes. These characteristics were evaluated by conducting various experiments such as physical inspection, moisture, impedance, swelling, color, texture, FTIR, microbiological, and sensory analysis. CL with different concentrations was incorporated into WWB formulations: P0.0 (0.0% *w*/*w* CL, control), P0.5 (0.5% *w*/*w* CL), P1.0 (1.0% *w*/*w* CL), P2.0 (2.0% *w*/*w* CL), and P3.0 (3.0% *w*/*w* CL). The inclusion of CL promoted the Maillard reaction (MR) compared to P0.0. The promotion of MR resulted in the formation of a shinier crust, which increased as the CL content was increased. P0.5 comprised large-sized pores and exhibited increased loaf height. CL-containing WWB formulations showed an increased moisture content and decreased impedance values compared to the control. FTIR analysis of P0.5 demonstrated the enhanced interaction and bonding of water molecules. P0.5 demonstrated optimal textural, colorimetric, and antimicrobial properties compared to other formulations. The sensory attributes of WWBs remain unchanged despite CL addition. In conclusion, P0.5 exhibited optimal characteristics associated with better quality and prolonged shelf life.

## 1. Introduction

Bread is considered a staple food across the globe as it is a significant source of dietary fiber, vitamins, micronutrients, and antioxidants [1]. It typically possesses a brownish crunchy crust and a soft crumb. A well-baked bread consists of a roasting aroma and great slicing qualities [2]. Flour, water, yeast, and salt (sodium chloride) are the fundamental ingredients for the process of breadmaking. When these four ingredients are properly combined, kneaded, fermented, and baked, it forms bread. The specific formulations can vary significantly based on the type of bread [3]. The flour quality is a crucial factor in bread making as it can greatly influence the texture, flavor, and overall quality of the bread [4]. Hence, the flour should be carefully selected based on the desired final product and its intended use. In general, white bread is one of the most commonly consumed types of bread worldwide. It is made with refined wheat flour and has a high glycemic index (GI). The high GI of white bread has been attributed to the presence of strongly gelatinized starch molecules, which are formed during the baking process at 250 °C. Salivary and pancreatic α-amylases can quickly break down the gelatinized starch molecules [5]. Due to this, refined wheat flour is rapidly digested and absorbed by the body, leading to a quick increase in blood glucose levels after consumption of white bread. Consequently, there is a spike in insulin levels to help regulate the elevated glucose levels [6]. Increased insulin levels have been associated with increased fat storage and reduced fat breakdown, thereby leading to obesity, cardiovascular diseases, and type 2 diabetes.

The aforementioned adverse effects of white bread can be minimized by consuming low-GI diets, which in turn promote better blood sugar control and healthier weight management [7]. Accordingly, whole-grain foods promote a slower glycemic response compared to refined grains. In this regard, whole wheat bread (WWB) is an excellent source of functional ingredients like essential amino acids, antioxidants, vitamins, and minerals. It is also rich in dietary fiber and phytochemicals [8]. Therefore, WWB is regarded as a great source of nutritional and functional elements for human health with numerous related advantages, such as a decrease in the risk of cardiovascular diseases, diabetes, cancer, and obesity. However, its use in food applications can be challenging due to some inherent characteristics that may affect product quality and sensory attributes. WWB has a lower bread volume, dense texture, elevated crumb hardness, and a nuttier flavor than white bread [9]. Also, due to the presence of bran and germ, yeast activity may be greatly hampered in WWB [10]. In this regard, the incorporation of specific fibers or polysaccharides in WWB has often been proposed to overcome these problems [11].

Polysaccharides (e.g., Arabic gum, sodium alginate, sesbania gum, chitosan, and water-soluble chitosan derivatives) are versatile ingredients that improve dough performance and enhance bread quality. The inclusion of polysaccharides can help provide a consistent crumb structure, softness, and improved sensory attributes of bread [12]. It has also been reported that polysaccharides also affect the bread volume by enhancing the viscosity of the batter. An increase in the viscosity of the batter reduces the gas diffusion rate and allows gas retention within the bread during baking. The authors also found that texture stability and moisture retention improved after polysaccharides were included in WWB. This, in turn, helps extend the shelf life of bakery products [13]. Accordingly, in a study by Tabara et al. (2016), the authors reported that the addition of Arabic gum to bread significantly decreased the process of staling by enhancing the formation of hydrophilic bonds between amylose and water molecules. Also, the incorporation of sodium alginate into wheat flour can result in improved breadmaking characteristics, including increased loaf height and specific volume [14]. Further, it has been found that sesbania gum, in combination with acetylated distarch adipate, can inhibit starch gelatinization [15]. One such promising polysaccharide is chitosan, which features many benefits and properties over the above-mentioned polysaccharides. The most prominent activity of chitosan is its microbiostatic activity, which can affect the shelf life of baked products. Additionally, chitosan also acts as a prebiotic and promotes health, with potential benefits in preventing coronary heart diseases, colon cancer, diabetes, and gastroenterological diseases [16].

Despite numerous advantages, the use of chitosan in various processes is limited due to its crystalline nature, which makes chitosan less hydrophilic. Hence, preparing its water-soluble derivatives has been proposed to eliminate this problem [17]. One such water-soluble derivative of chitosan is chitosan lactate (CL), a linear copolymer comprising units of glucosamine and *N*-acetyl glucosamine. CL has been employed to enhance the quality and shelf life of bread [18]. CL has numerous applications in food preservation and packaging, attributed to its notable features such as moisture retention capacity, antibacterial activity, and effectiveness in disease prevention [19]. Research by Rakcejeva et al. (2011) indicated that the inclusion of chitooligosaccharide lactate in bread formulations influences the firmness of the bread, leading to a better texture and structural integrity [20]. However, to the best of our knowledge, there are no articles on the alteration in the properties of WWB after incorporating CL. Hence, there is a need to examine the effect of CL on the properties of WWB. Considering the advantages of CL, our study has been conducted to assess the impact of incorporating CL at various concentrations (0%, 0.5%, 1%, 2%, and 3% *w*/*w*) on the properties of WWB. Researchers have reported that adding polysaccharides in 1% to 3% (*w*/*w*) concentrations provides the bread with optimum quality, such as low proof times, strong resistance to extension, beneficial moisture content, and high specific bread volume with soft crumbs [21]. Hence, we preferred the addition of CL in WWB, wherein the CL was added in the concentration range of 0%, 0.5%, 1%, 2%, and 3% (*w*/*w*). The bread with 0% CL was used as the control. Various characterizations of the prepared bread samples were performed by visual and physical inspection, moisture content determination, color analysis, microscopy, impedance analysis, microbiological analysis, texture analysis, FTIR spectroscopy, swelling study, and sensory analysis to evaluate the impact of varying concentrations of CL on the WWB formulations.

## 2. Materials and Methods

### 2.1. Materials

WWF (Brand: Aashirvaad, Make: ITC Limited in Kolkata, India), common salt (Brand: Tata, Make: Tata Chemicals Ltd., Mumbai, India), sugar, rice bran oil (RBO) (Brand: Fortune, Make: Fortune Oil Company, Ahmedabad, India), and instant dry yeast (Make: Goodrich Carbohydrates Ltd., Nagla Megha, India) were purchased from the local market. As per the information presented on the packaging label, the nutritional content of WWF per 100 g is 76.8 g of carbohydrates, 10.5 g of protein, 340 kCal of energy, 2.3 g of sodium, and 1.4 g of total fat. The nutritional content of RBO is 34 g of polyunsaturated fatty acid, 24 g of saturated fatty acid, 40 g of mono-unsaturated fatty acid, and 2 g of trans fatty acid. Additionally, RBO contains 50 mg of vitamin E and 1000 mg of γ-oryzanol. Chitosan lactate (molecular weight: 43.506 kDa; degree of deacetylation: 90% [22]; viscosity: 109.08 cps [23]) was procured from Everest Biotech, Bangalore, India. The viscosity and molecular weight of chitosan lactate were determined by the Ostwald viscometer. Nutrient agar and sodium chloride were procured from HiMedia Laboratories Pvt. Ltd., Mumbai, India, and Loba Chemie Pvt. Ltd., Mumbai, India, respectively.

### 2.2. Preparation of WWBs

WWB samples containing varying CL concentrations, ranging from 0%, 0.5%, 1%, 2%, and 3%, were prepared. The samples were denoted as P0.0 (control; 0% *w*/*w* of CL), P0.5 (0.5% *w*/*w* of CL), P1.0 (1% *w*/*w* of CL), P2.0 (2% *w*/*w* of CL), and P3.0 (3% *w*/*w* of CL). The components listed in (Table 1) were mixed and placed inside the baking pan of the breadmaking machine (Make: Kent RO Systems Ltd., Noida, India), which was set to the “whole wheat bread” program, which started with a heating period of 5 min. Then, the mixture underwent a simultaneous kneading and resting process within the machine for 1 h, followed by a 2 h fermentation period at 30 °C. After fermentation, the dough was transferred to a loaf tin and evenly spread across the surface using a bread spreader. Subsequently, the loaf tin was placed inside a microwave oven preheated for 5 min at 180 °C. The dough was then baked at 180 °C for a total duration of 36 min. After the baking process, the prepared WWBs were cooled for 1 h at room temperature (25 °C).

### 2.3. Moisture Content Analysis

A digital moisture analyzer (Model: PGB1MB analyzer; Make: Wensar, Chennai, India) equipped with a halogen heating source was employed to determine the moisture content of the WWB samples. The WWB samples were cut into small pieces to facilitate complete moisture evaporation. The sample pieces (~2 g) were then kept in the aluminum pan, and the initial weight was recorded. Then, the temperature of the moisture analyzer was set at 180 °C. The loss in the weight of the samples was monitored as the moisture loss from the samples. This procedure was continued until the constant weight was achieved [24].

### 2.4. Impedance Analysis

The electrical profile of the WWB samples was determined using an impedance analyzer (Model: Impedance breakout board for Analog Discovery 2, Digilent, National Instrument, Austin, TX, USA). Impedance spectroscopy was conducted using a set of circular stainless-steel probes (diameter: 10 mm) positioned at a separating distance of 1 cm. A cuboid crumb portion of WWB (dimensions: 4 cm (L) × 3 cm (B) × 4 cm (H)) was cut. Then, the electrode system was inserted into the crumb. Subsequently, the impedance profile was recorded in the frequency range of 1 Hz and 1 KHz. Throughout the measurement process, a resistor of 1 MΩ was employed as a reference for the analysis [25].

### 2.5. Swelling Study

The swelling study of WWB samples was conducted in triplicates. For this purpose, the crumb section of the WWB was initially cut into small cubes (2 cm × 2 cm), whose weights were nearly equal. Subsequently, these cubes were placed into 100 mL plastic beakers, and the beakers were positioned in a water bath (Model: Lmwb-8, Labman Scientific Instruments Pvt. Ltd., Chennai, India), maintained at 37 °C. The weights of the samples were recorded over a period of 5 h., which were then employed to compute the swelling index (Equation (1)).

(1) $$SI\;(\%)=\frac{w_{F}-w_{I}}{w_{I}}$$

where *SI* (%) is the swelling index, *W_F_* is the final weight (after the swelling process), and *W_I_* is the initial weight (before the swelling process) of the WWB samples.

### 2.6. FTIR Analysis

The IR absorption spectra of the WWB formulations were acquired using an ATR-FTIR spectrophotometer (Model: Alpha-E; Bruker, Bremen, Germany) equipped with an attenuated total reflectance (ATR) module containing a Zinc selenide (ZnSe) crystal. Spectra of the WWB formulations were recorded over the range of 4000–500 cm^−1^, with each sample subjected to 25 scans at a spectral resolution of 4 cm^−1^. Following the baseline correction, the FTIR spectrum was deconvoluted with Gaussian curves using the “Multiple Peak Fit” tool and the Levenberg–Marquardt method in the Origin Pro software package (v9.1, Northampton, MA, USA). Numerical deconvolutions of the OH band (3800–3000 cm^−1^) and the band corresponding to the starch region (1070–950 cm^−1^) were performed. R^2^ > 0.99 and χ^2^ < 0.001 were the fundamental elements considered during the deconvolution process. Sub-bands associated with specific interaction/conformation were added and divided by the total area of the corresponding main band. Subsequently, the percentage contribution of the specific sub-bands, corresponding to specific interaction or conformation, to the overall area was calculated [26].

### 2.7. Colorimetric Analysis

The colorimetry study for WWB samples was performed using a laboratory-developed colorimeter to obtain CIELab (L*, a*, and b*) values, whose details have been reported by our group in [27]. Firstly, the colorimeter was calibrated with the help of white and black tiles. The bread sample (square-shaped; dimension: 2 cm × 2 cm) was kept in a 35 mm Petri dish and then subjected to colorimetric evaluation to acquire the L* (lightness), a* (red to green), and b* (yellow to blue) color parameters. Subsequently, the whiteness index (WI) [25], yellowness index (YI) [25], and brownness index (BI) [28] of the WWB samples were calculated from the obtained L*, a*, and b* data using Equations (2)–(4), respectively.

(2) $$WI=100-\sqrt{{\left(100-L*\right)}^{2}+{\left(a*\right)}^{2}+{\left(b*\right)}^{2}}$$

(3) $$YI=142.86\times \frac{b*}{L*}$$

(4) $$BI=\frac{\left\{100\left(X-0.31\right)\right\}}{0.17}$$

$$X=\frac{\left(a*+1.75L*\right)}{\left(5.645\times L*+a*-3.012b*\right)}$$

where L*, a*, and b* are the color parameters of the CIELab system. *WI* is the whiteness index, *YI* is the yellowness index, and *BI* is the brownness index.

### 2.8. Microscopic Analysis

The surface topology of the crust and crumb of the WWB samples was observed using a Stereo Zoom Microscope (Model: SM-2TZ; Make: AMscope, Irvine, CA, USA). WWB samples were cut to have a square surface with a dimension of 2 cm × 2 cm. The microscope was attached with an external eyepiece camera (AMscope MD500, Irvine, CA, USA) to capture microscopic images [25].

### 2.9. Texture Analysis

Texture analysis of WWB samples was conducted to determine the textural and viscoelastic attributes.

#### 2.9.1. Texture Profile Analysis

Texture profile analysis was performed to evaluate different textural characteristics like hardness, chewiness, springiness, gumminess, cohesiveness, and resilience. The WWB formulations were cut into small cubes 2 cm in width. These cubes were subjected to the texture analyzer (Model: Texture analyzer HD plus; Stable Micro Systems, Godalming, UK) for texture profile analysis. Two compression cycles at a rate of 1 mm/s were used to deliver a 50% strain to the WWBs samples using a flat probe with a diameter of 65 mm. There was a 5 sec break maintained between each of the two cycles [25].

#### 2.9.2. Stress Relaxation Profile

The stress relaxation profile is a technique for characterizing the viscoelastic properties of the WWB formulations. In this experiment, a texture analyzer (Model: Texture analyzer HD plus; Stable Micro Systems, Godalming, UK) was used. The WWB formulations were prepared in cuboidal shapes with sides measuring 2 cm. A flat probe with a diameter of 63.5 mm was employed to conduct the stress relaxation profile. This probe compressed the WWB cubes to a depth of 2 mm at a rate of 1 mm/s, activated by a trigger force of 5 g. This constrained state was maintained for 60 s, during which the relaxation profiles were generated by monitoring the force values over time. Following this, the probe was retracted to its initial position [25].

### 2.10. Microbiological Analysis

#### 2.10.1. Preparation of Media and Reagents

Initially, the nutrient agar (HiMedia Laboratories Pvt. Ltd., Mumbai, India) (2% *w*/*w* in water), Petri plates, test tubes, and saline solution were autoclaved (121 °C and 15 PSI at 15 min). Then, stock solutions for the prepared WWB formulation (after baking) were made by cutting the formulations into cubes (2 cm × 2 cm × 2 cm) and were stored in sterile air-tight containers. Then, 1 g of the formulations was placed in 9 mL of sterile saline solution (0.85% sodium chloride solution) to prepare the stock solution. These stock solutions were then vortexed for 5 min to achieve a homogenized state followed by ten-fold dilutions of stock solution using sterile saline solution (10^−1^ to 10^−3^).

Nutrient agar plates were prepared by pouring 25 mL of molten nutrient agar (maintained at 40 °C) into sterile Petri plates, which were then allowed to solidify for 45 min at room temperature (25 °C).

#### 2.10.2. Estimation of the Total Viable Count (TVC)

Microbial analysis of the prepared WWB formulations was conducted using the culture method using nutrient agar as the growth medium. In the nutrient agar plates, 0.1 mL of each dilution was placed on the surface of the solidified media and evenly spread. These plates were then incubated overnight at 37 °C. This experiment was conducted in triplicate, with each trial conducted on alternate days. The total viable count (TVC) of bacteria was expressed as CFU (colony-forming units) per gram of the WWB formulations.

### 2.11. Sensory Analysis

The sensory assessment of the prepared WWB formulations was conducted with 20 semi-trained panelists, comprising students and PhD scholars aged between 20 and 35 years, who evaluated the WWB samples blindfolded. Initially, the WWB formulations were sliced, and each bread slice was subjected to sensory analysis by the panelists. Panelists rated the WWB formulations based on their satisfaction level using a scale of 1 to 5, where 1 represented extreme dislike, 2 indicated dislike, 3 denoted neither like nor dislike, 4 signified like, and 5 represented extremely like. The sensory attributes considered included appearance, color, aroma, softness, and overall impression of the WWB formulations.

### 2.12. Statistical Analysis

The experiments were performed in triplicate, and the results were expressed as the average ± standard deviation. SPSS Statistics software (ver. 28, IBM Inc., Chicago, IL, USA) was used for statistical analysis. The significant difference between the values was detected using ANOVA (one-way variance analysis), followed by the Tukey post hoc test. The level of significance (*p* < 0.05) was used to find significant differences between the values.

## 3. Results

### 3.1. Visual and Physical Inspection

**Figure 1:**
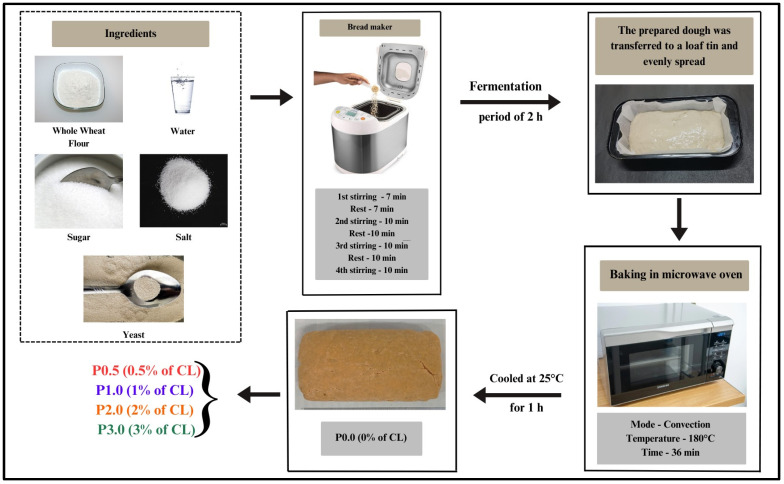
Flowchart illustrating the mechanism of WWB (whole wheat bread) preparation.

**Figure 2:**
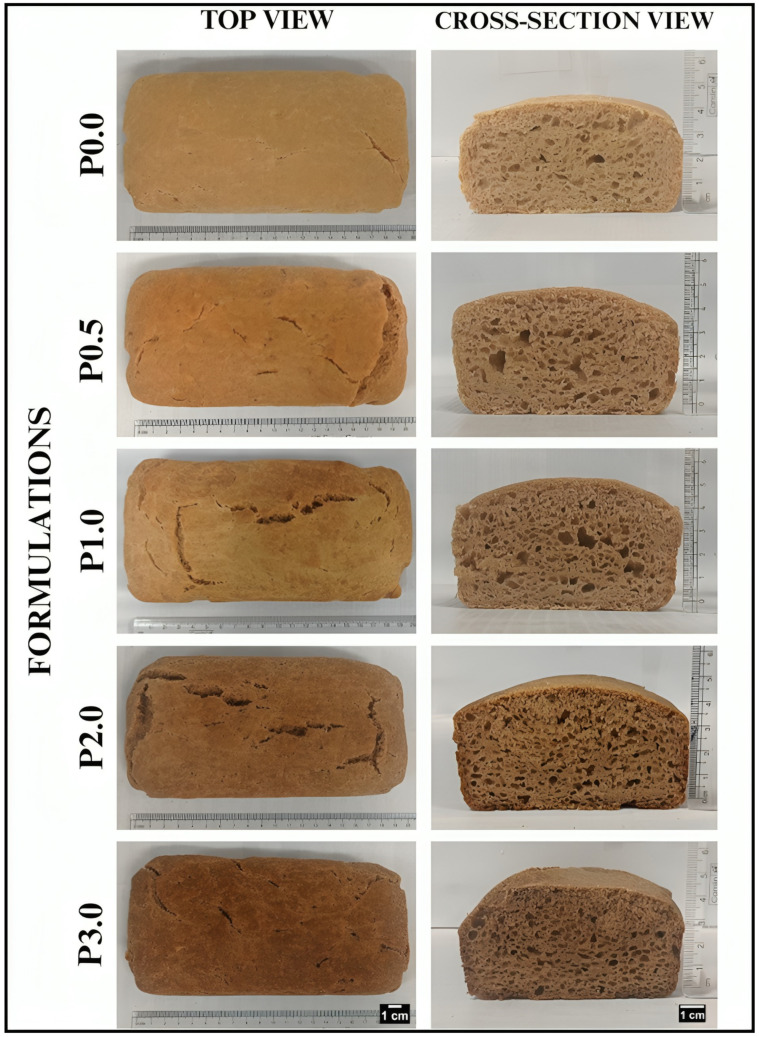
Top view and cross-section view of whole wheat bread (WWB) formulations containing chitosan lactate (CL) in different concentrations: P0.0 (control, absence of CL), P0.5, P1.0. P2.0 and P3.0 (0.5%, 1.0%, 2.0%, and 3.0% of CL).

The WWB samples were allowed to cool down for 1 h at 25 °C after baking. This brought the WWB samples to a comfortable temperature for handling and performing experiments. Visually, P0.0 displayed a yellowish-brown color due to lutein, a vital carotenoid in whole wheat (Figure 1) [29]. Further, the formulations exhibited a sequential rise in brownness from P0.5 to P3.0, which can be attributed to the Maillard reaction (Figure 2) [30]. In the initial phase of the Maillard reaction, chitosan derivatives form complexes with glucose, resulting in the formation of chitosan–glucose conjugates. At high temperatures, these conjugates form a brown pigment called melanoidin [31]. The increasing concentration of CL in WWB samples must have contributed to the formation of more CL-glucose complexes. This promoted the Maillard reaction, leading to a progressively darker color of WWB samples as the CL content was increased.

The cross-section view of bread samples provides information about the porosity of bread (Figure 2). P0.0 had a reduced number of pores compared to all other formulations. Large-sized pores were found in P0.5 and P1.0. However, an uneven distribution of pores was observed in P2.0. Interestingly, when the CL content was increased to 3% (*w*/*w*) in P3.0, an even distribution of smaller pores was observed. The porous structure(s) of the WWB samples have been related to the improved yeast activity, thereby causing improved fermentation [32]. This suggests that the presence of CL promoted fermentation in the bread samples. Further, the interaction of chitosan and its derivatives with the gluten network has also been associated with the porosity of wheat bread. Overall, we found that P0.5, a formulation with 0.5% (*w*/*w*) of CL, exhibited comparatively larger pore size than the other bread formulations. This suggests better facilitation of the fermentation process in P0.5, which consequently leads to enhanced gas-holding capacity and stability of the bread cells [33]. Similar observations have been reported by X. Dou et al. (2024), where the authors added 0.5% of chitosan hydrochloride to the bread [34].

**Figure 3:**
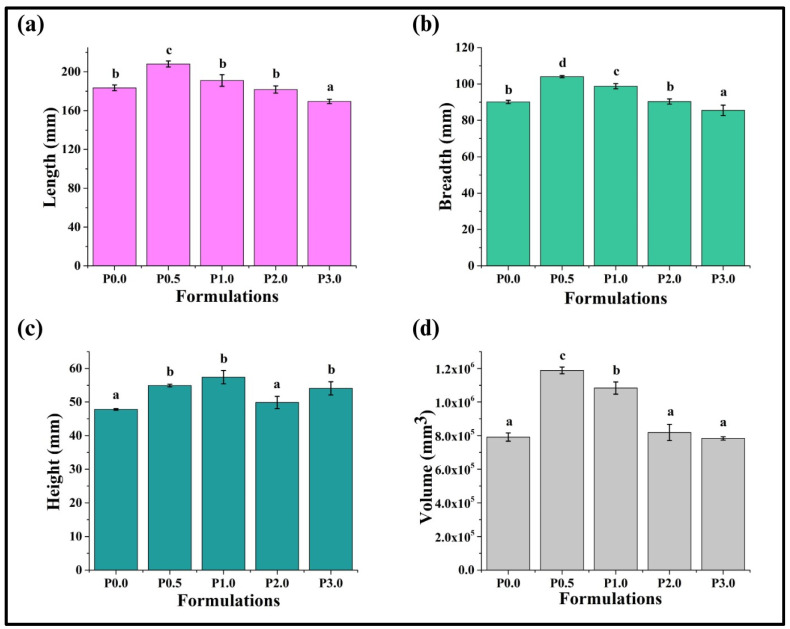
Physical dimensions of whole wheat bread (WWB) formulations containing chitosan lactate (CL) in different concentrations: P0.0 (control, absence of CL), P0.5, P1.0. P2.0, and P3.0 (0.5%, 1.0%, 2.0%, and 3.0% of CL). (**a**) Length, (**b**) breadth, (**c**) height, and (**d**) volume. The values denoted in the graphs are the average ± SD of triplicate samples. Bars with different letters differ significantly (*p* < 0.05).

The physical inspection of bread is a critical factor in determining its quality and overall appeal. Some physical characteristics like loaf volume, porosity, and hardness are key parameters that are used to determine the palatability of bread [35]. It was found that the length of P0.0 was 183.49 ± 3.03 mm. In P0.5, there was a significant rise in length (208.02 ± 3.13 mm; *p* < 0.05). Further addition of CL reduced the length values of P1.0, P2.0, and P3.0, respectively. However, the lengths of P1.0 and P2.0 were statistically similar (*p* > 0.05) (Figure 3). The breadth of the prepared WWB samples value also followed a similar pattern to that of the length. In short, the breadth of P0.5 was the highest, while the breadth of P3.0 was the lowest. The height of P0.0 was 47.82 ± 0.24 mm. It was observed that the heights of P0.5, P1.0, and P3.0 were greater than P0.0 (*p* < 0.05), whereas the height of P2.0 was similar to that of the control (P0.0) sample (*p* > 0.05). The increase in the heights of P0.5 and P1.0 can be attributed to the presence of larger pores, while the even distribution of small pores can be reasoned for the enhanced height of P3.0, as was observed from the cross-section view. This suggests that the fermentation process was promoted in P0.5, P1.0, and P3.0 [33].

The length, breadth, and height information of the WWB formulations were used to calculate the estimated volume. It was found that the volume of P0.0 was 7.90 × 10^5^ ± 0.24 × 10^5^ mm^3^. The addition of CL in the lowest amount in P0.5 (0.5% of CL) exhibited the highest volume compared to all other formulations (*p* < 0.05). With a subsequent increase in the CL content, there was a reduction in the volume of the WWB formulations in P1.0, P2.0, and P3.0, respectively. The volumes of P0.0, P2.0, and P3.0 were statistically similar (*p* > 0.05). An alteration in the bread volumes in the presence of chitosan derivatives has been associated with altered water molecule distribution and enhanced drying of the starch–gluten network [36]. In the study by X. Dou et al. (2024), it has been reported that the addition of 0.5% of the chitosan derivative led to an improved loaf volume, which is concurrent with the results obtained in our study wherein the highest loaf volume was observed in P0.5.

### 3.2. Moisture Content Analysis

**Figure 4:**
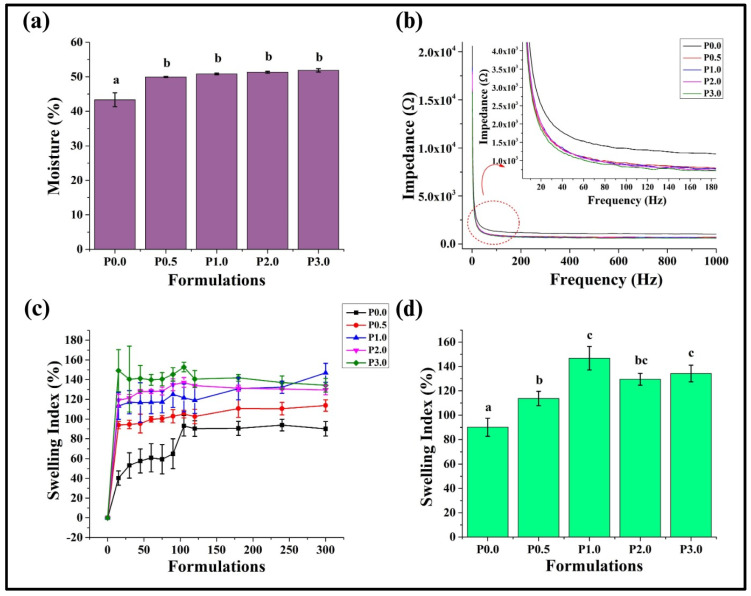
Graph representing: (**a**) moisture content, (**b**) impedance analysis (the red dotted circle represents the area magnified), (**c**) swelling index (%) of whole wheat bread (WWB) formulations, (**d**) swelling index (%) of WWB formulations at the end of the study (5 h). WWB formulations containing chitosan lactate (CL) in different concentrations: P0.0 (control, absence of CL), P0.5, P1.0, P2.0, and P3.0 (0.5%, 1.0%, 2.0%, and 3.0% of CL). The values denoted in the graphs are the average ± SD of triplicate samples. Bars with different letters differ significantly (*p* < 0.05).

The sensory characteristics of bread, such as flavor, freshness, volume, texture, color, and overall acceptability, are notably influenced by moisture content. It is well documented that the amount and distribution of water in bread play a critical role in governing its shelf life, susceptibility to microbial spoilage, crumb softness, crust crispness, and overall tendency to stale [37]. Therefore, it has become customary to determine the moisture content of WWB formulations. Moisture analysis of WWB samples revealed that P0.0 showed the lowest moisture content (43.33 ± 2.01%) compared to the CL-containing bread formulations (*p* < 0.05) (Figure 4a). The subsequent addition of CL in the WWB samples resulted in a significant rise in the moisture content of the P0.5 sample (*p* < 0.05). Further addition of CL did not result in significant changes in the moisture content of P1.0, P2.0, and P3.0 compared to P0.5 (*p* > 0.05). The increase in moisture content of WWB formulations due to the addition of CL can be attributed to the higher solubility of CL in water. This can be reasoned by the presence of lactate molecules, which enhances the hydrophilic character of CL [38]. The higher moisture content in bread with a lower concentration of CL can be attributed to the ability of CL to retain water. Further addition of CL in bread might have resulted in the saturation of the water-holding capacity, thereby resulting in no further rise in the moisture content of bread [18]. This can explain the insignificant variation in the moisture content in P1.0, P2.0, and P3.0.

### 3.3. Impedance Analysis

Electrical impedance spectroscopy (EIS) is a method employed to determine the electrical characteristics of materials by recording the changes in impedance values induced by varying the frequency of an applied voltage. The impedance profile of WWB formulations was measured at frequencies ranging from 1 Hz to 1 KHz. At lower frequencies, from 1 Hz to 100 Hz, P0.0 had the highest impedance, whereas P3.0 had the lowest impedance. P0.5, P1.0, and P2.0 showed overlapping impedance patterns throughout the frequency range (Figure 4b). At higher frequencies (100 Hz to 1 KHz), where the impedance values reached a basal level, the order of the impedance values remained the same as that in lower frequencies. A similar study was conducted by Kertesz et al. (2015), in which the authors reported an inverse relationship of impedance with that of moisture content, pore size, and pore density [39]. P0.0 had a higher impedance value due to its smaller pores and low moisture content (Figure 4a). In contrast, the other WWB formulations with CL (P0.5, P1.0, P2.0, and P3.0) exhibited higher moisture content, resulting in lower impedance values than the control. The presence of larger-sized pores with uniform distribution in P0.5 (Figure 2) might have facilitated better movement of water molecules within the breadcrumb, thus resulting in a lower impedance value.

### 3.4. Swelling Study

The swelling study is crucial in assessing bread quality, indicating its capacity to absorb and hold moisture. This characteristic directly influences the texture, crumb structure, and overall quality of the bread [40]. A swelling study was performed for WWB formulations for the duration of 5 h. It was noted that P0.0 exhibited the lowest swelling index (SI) during the course of the experiment. The inclusion of CL in WWB formulations resulted in a significant increase in the SI value (Figure 4c). The observed results can be explained by the study carried out by Kowalczyk et al. (2023). In the study, the authors reported that chitosan molecules functionalized with lactic acid have better moisture-retention capacity, thereby rendering it highly beneficial for applications with higher moisture absorption [41]. Among the CL-containing formulations, P0.5 showed the lowest SI, while the SI of the other formulations was similarly valued (Figure 4d) (*p* > 0.05). It has been previously reported that the water-holding capacity of chitosan derivatives is better at lower concentrations, while a higher concentration of chitosan derivatives can lead to saturation of its water-holding capacity. This can result in no further increase in the water-holding capacity of the formulations, even with a higher chitosan derivative content [18]. This can clearly describe the variation in the SI values of CL-laden WWB formulations.

### 3.5. FTIR Analysis

**Figure 5:**
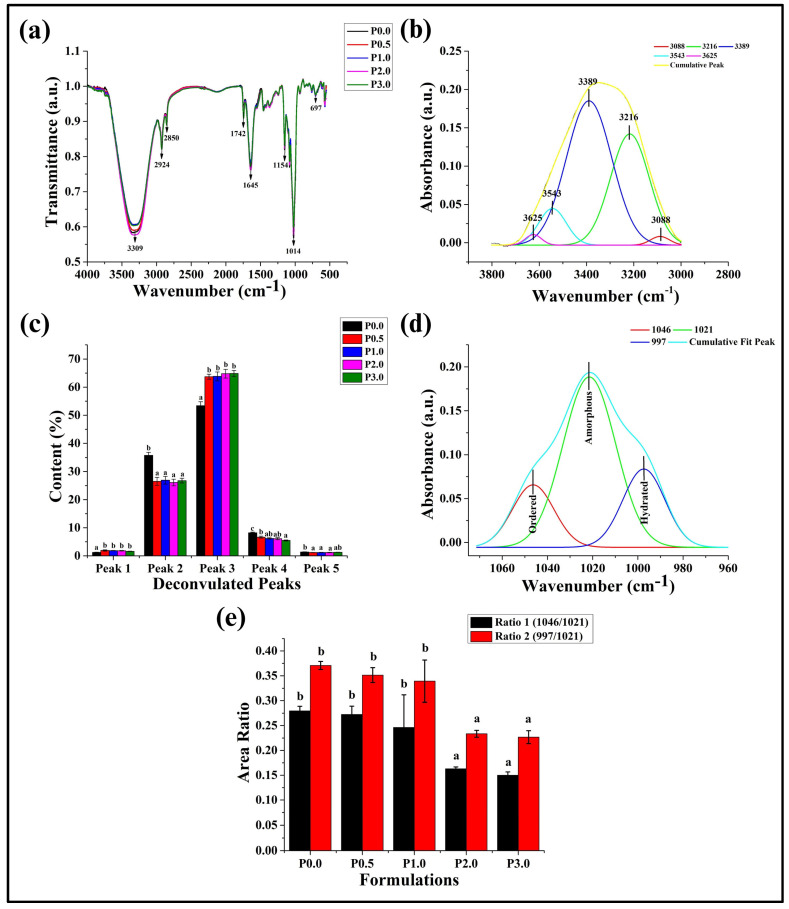
(**a**) Fourier Transform Infrared Spectroscopy (FTIR) spectra of whole wheat bread (WWB) formulations, (**b**) Deconvulated bands of the water region (3800 to 3000 cm^−1^) of WWB formulations, (**c**) Bar graph representing the area under the curve (AUC) of the peaks found in the water region, (**d**) Deconvulated bands of the starch fingerprint region (1070–950 cm^−1^), and (**e**) Bar graph representing the area ratio of the starch region in the WWB formulations. WWB formulations containing chitosan lactate (CL) in different concentrations: P0.0 (control, absence of CL), P0.5, P1.0, P2.0, and P3.0 (0.5%, 1.0%, 2.0%, and 3.0% of CL). The values denoted in the graphs are the average ± SD of triplicate samples. Bars with different letters differ significantly (*p* < 0.05).

Fourier-transform infrared (FTIR) spectroscopy plays a crucial role in exploring both the chemical and structural properties of bread, which can describe the interaction between the functional groups of components present in the food products. It also helps to analyze the development of resistant starch during the fermentation process [42]. After analyzing the FTIR spectra, it was found that the major peaks appeared at 3309, 2924, 2850, 1742, 1645, 1154, 1014, 697, and 571 cm^−1^ (Figure 5a). The broad peak at 3309 cm^−1^ is associated with the stretching vibration of the hydroxyl (OH) groups. This peak can describe the moisture content of WWB formulations [43]. Further, the two major peaks at 2924 cm^−1^ and 2850 cm^−1^ usually correspond to the stretching of CH groups [44]. The peak at ~1600 cm^−1^ can be ascribed to C=O stretching, and those between 1500 cm^−1^ and 650 cm^−1^ are associated with C-O-C stretching and CH bending, respectively [43].

The variations in the strength of hydrogen bonds between proteins and water molecules, along with the amount of free water present inside the gluten network, can also be investigated with the help of FTIR bands [45]. Hence, the deconvolution of the water region (3800 to 3000 cm^−1^) on the FTIR spectra of the WWB formulations was performed. The O-H band consists of five overlapping bands situated at 3090, 3220, 3393, 3540, and 3625 cm^−1^. It has been reported earlier that the peaks at the higher wavenumbers (3540 cm^−1^ and 3625 cm^−1^) are associated with the molecules that specifically participate in water–water interactions. In contrast, the peaks at lower wavenumbers (3090 cm^−1^, 3220 cm^−1^, and 3393 cm^−1^) are linked to the molecules that form strong H-bonds [46]. The peak at 3090 cm^−1^ can be attributed to the O-H vibration of a strong hydrogen-bonded water structure associated with the Fermi resonance of the overtone of O-H plane bending. Further, strong O-H interactions of molecules can give rise to the peak at 3220 cm^−1^ [47]. The peak at ~3390 cm^−1^ is attributed to the interaction of weakly bound water molecules, and the peaks at ~3540 cm^−1^ and ~3620 cm^−1^ are associated with symmetric and asymmetric O-H vibrations of free water or non-hydrogen-bonded molecules, respectively. In our case, the deconvolution of the water region resulted in five distinct peaks, whose positions were at 3088 cm^−1^ (peak 1), 3216 cm^−1^ (peak 2), 3389 cm^−1^ (peak 3), 3543 cm^−1^ (peak 4), and 3625 cm^−1^ (peak 5) (Figure 5b). Peak 2 (3216 cm^−1^) and Peak 3 (3389 cm^−1^) were dominant among all the 5 peaks.

Peak 1 is associated with the Fermi resonance of the overtone of O-H plane bending along with O-H vibrations [47]. The area under the curve (AUC) value of P0.0 was the lowest compared to other formulations (*p* < 0.05). In contrast, the AUC values of the rest of the formulations were statistically similar (*p* > 0.05) for peak 1 (Figure 4c). Peak 2 represents the strongly bonded water molecules. In addition to that, it also attains Fermi resonance with the O-H vibration of strongly bonded water molecules. P0.0 showed a higher AUC value for peak 2 (*p* < 0.05), while all other WWB formulations were comparable (*p* > 0.05). Further, peak 3 corresponds to weakly bonded water molecules. P0.0 exhibited the lowest AUC value for peak 3 compared to other formulations. The AUC of peak 3 for P0.5, P1.0, P2.0, and P3.0 were statistically similar (*p* > 0.05). Peak 4 is associated with the symmetric O-H vibrations of free water molecules. A decreasing trend in the AUC values of WWB formulations was observed for peak 4. Peak 5 corresponds to the asymmetric O-H vibrations of water molecules, which are freely present in the gluten network of the bread. The AUC values of WWB formulations were comparable for peak 5 (*p* > 0.05).

The above observations suggest that the addition of CL in the WWB formulations might have improved the bonding between water molecules and proteins. In CL-containing formulations, the content (%) of strongly bonded water molecules (peak 1) and weakly bonded water molecules (peak 3) were higher (Figure 5c). In addition to that, the number of free water molecules decreased from P0.0 to P3.0 in a significant manner (*p* < 0.05). From the above results, it can be concluded that the addition of CL might have improved the bonding of water molecules.

A study by Pulatsu et al. (2021) reported that the fingerprint region of starch, which indicates its molecular organization, is found in the spectra ranging from 1070 cm^−1^ to 950 cm^−1^ [48]. Accordingly, the deconvolution of the spectra in the 1070–950 cm^−1^ wavenumber region was performed. Three overlapping bands located at 1046 cm^−1^, 1021 cm^−1^, and 997 cm^−1^ linked to the ordered, amorphous, and hydrated crystalline structures of starch, respectively, were observed (Figure 5d) [48]. The alignment of helices inside the short-range order of starch can be identified by the ratio of the absorption peaks at 1046 cm^−1^ to 1021 cm^−1^ (ratio 1). The presence of water molecules that are bound to the starch molecules can affect this alignment. Further, the ratio of the absorption peaks at 997 cm^−1^ to 1021 cm^−1^ (ratio 2) has been used to assess the short-range structure and the existence of double helices in starch [49,50]. In the case of ratio 1, P0.0, P0.5, and P1.0 exhibited statistically similar AUC values (*p* > 0.05). The ratio 1 values of P2.0 and P3.0 were lower than P0.0, P0.5, and P1.0 (*p* < 0.05). Among P2.0 and P3.0, the ratio 1 values were comparable (*p* > 0.05) (Figure 5e). The reduction in ratio 1 indicates the changes in the alignment of starch helices. It can be explained by the binding of chitosan derivatives with the starch chains, which can induce steric hindrances. This consequently prevents the tight packaging of starch helices, thereby reducing the alignment ratio [51]. Ratio 2 followed a similar trend of AUC values as in ratio 1. The reduction in the ordered or crystalline structure of starch can be attributed to the addition of chitosan derivatives. This can also be associated with the introduction of steric hindrance in starch molecules after the addition of CL, resulting in the formation of damaged starch molecules and, consequently, decreasing the ordered structure of helices in starch [51].

### 3.6. Colorimetric Analysis

**Figure 6:**
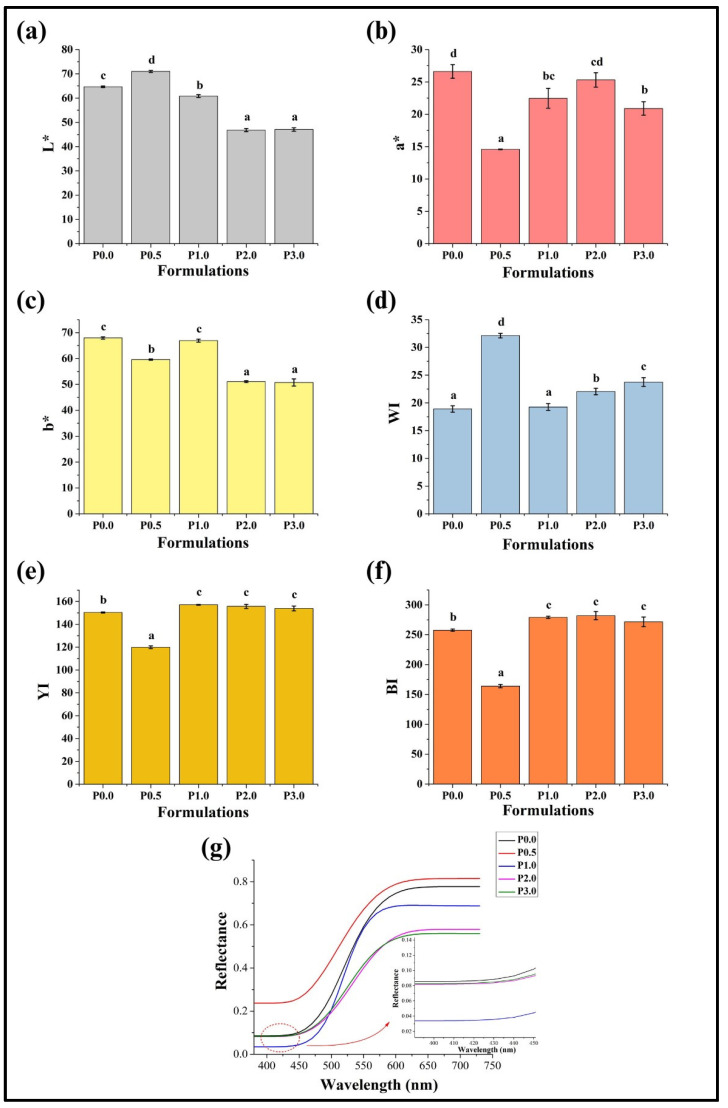
Graphs representing the colorimetric analysis of whole wheat bread (WWB) formulations: (**a**) L*, (**b**) a* (**c**) b*, (**d**) WI, (**e**) YI, (**f**) BI, of WWB formulations. (**g**) Line graph representing the reflectance of WWB samples against the visible spectrum (the red dotted circle represents the area magnified). WWB formulations containing chitosan lactate (CL) in different concentrations: P0.0 (control, absence of CL), P0.5, P1.0, P2.0, and P3.0 (0.5%, 1.0%, 2.0%, and 3.0% of CL). The values denoted in the graphs are the average ± SD of triplicate samples. Bars with different letters differ significantly (*p* < 0.05).

Colorimetric analysis is a crucial study that helps characterize the sensory qualities of food products. In this study, the CIELab (L*, a*, and b*) color values were used to analyze the color of the prepared bread formulations [52]. L* indicates the variation in the gray level values that is related to the brightness or luminosity of the samples. Its value lies between 0 and 100, corresponding to a black and white color, respectively. In contrast, the a* and b* values range from −120° to +120°, considered chromatic components within the CIELab color system. The positive a* axis exhibits redness, while the negative a* axis indicates a green color. Similarly, the negative b* axis displays a shift towards a blue color, whereas a yellow shade appears along the positive b* axis [53]. After performing the colorimetric analysis, the average L* values of the WWB samples ranged between 46 and 70 (Figure 6a). The L* value of P0.0 was 64.64 ± 0.32, and after the incorporation of CL in the WWB sample, a significant rise was observed in the P0.5 (70.98 ± 0.42) (*p* < 0.05). Thereafter, there was a monotonous reduction in L* values of P1.0, P2.0, and P3.0, respectively, as the CL content was increased. The L* values of P2.0 and P3.0 were comparable (*p* > 0.05). This observation of reduced L* values with the increase in the CL content can be reasoned for the Maillard reaction. It has been previously reported that the Maillard reaction plays a crucial role in the development of color in bakery products, including WWBs, and can explain the darker color of WWB formulations when the CL content was increased [54]. The average a* values of the WWB samples ranged between 14 and 26 (Figure 6b). It was observed that the a* value of P0.0 was 26.61° ± 1.05°, suggesting the presence of a reddish hue in P0.0. This red hue is due to carotenoids and anthocyanins present in the bran of the whole wheat [55]. P0.5 exhibited the lowest a* value (*p* < 0.05), followed by an elevation in a* value in P1.0 (*p* < 0.05). Furthermore, there was no noticeable change in the a* components of P1.0, P2.0, or P3.0. When the whole wheat flour was replaced with CL in P0.5, P1.0, P2.0, and P3.0, the variation in a* values can be reasoned to the Maillard reaction [37]. The average values of the b* component of the WWB formulations lie between 50 and 67 (Figure 6c). P0.0 and P1.0 exhibited higher b* values than the other formulations (P0.5, P2.0, and P3.0) (*p* < 0.05). P0.5, which contained the lowest amount of CL, exhibited a moderate b* value. P2.0 and P3.0 showed the lowest b* values compared to other formulations (*p* < 0.05). The yellow shade in the WWB samples can be attributed to the presence of lutein content [29].

The above L*, a*, and b* values were used to determine the whiteness index (WI), yellowness index (YI), and brownness index (BI). The WI of P0.0 was found to be 18.9 ± 0.56. P0.5 showed the highest WI when compared to all the formulations (*p* < 0.05), which was followed by a decline in the WI value of P1.0 (Figure 6d). The WI value of P1.0 was similar to that of the control (P0.0) (*p* > 0.05). Further addition of CL in WWB formulations resulted in an increasing trend in WI values of P2.0 and P3.0 (*p* < 0.05). The WI values of WWB formulations can be associated with the inverse relationship between WI and YI of the bread [56]. The YI value of P0.0 was 150.35 ± 0.50 (Figure 6e). There was a decrease in the YI value in P0.5, the lowest compared to all other formulations (*p* < 0.05). P1.0, P2.0, and P3.0 exhibited higher YI values compared to P0.0 and P0.5. The variations in the YI values of P1.0, P2.0, and P3.0 exhibited no significant differences (*p* > 0.05). The presence of lutein can be reasoned for the YI of the WWB formulations [29]. P0.0 exhibited a moderate BI value (257.54 ± 1.97) (Figure 6f). The BI value of P0.5 was the lowest of all the formulations (*p* < 0.05). Subsequently, further addition of CL led to an increase in the BI values, which were higher than the BI values of P0.0 and P0.5. The BI values of P1.0, P2.0, and P3.0 were statistically similar (*p* > 0.05). The brownness index of WWB formulations can be attributed to the Maillard reaction [37]. The BI values of all the formulations were much higher than the WI, and the YI values suggested a predominant brownish hue.

Reflectance analysis plays an important role in studying the characteristics of bread during development and baking. The final color of the bread crust and crumb provides a reliable indication of overall bread freshness, quality, and flavor. Bread color is a key attribute influencing consumer perception and purchase decisions [57]. P0.0 exhibited moderate reflectance, and P0.5 showed the highest reflectance throughout the visible spectrum range (Figure 6g). P1.0 exhibited the lowest reflectance at lower wavelengths (400 nm to 500 nm), but an elevation in the reflectance value of P1.0 was observed at higher wavelengths (500 nm to 730 nm). Reflectance values of P2.0 and P3.0 were found to be in overlapping patterns at lower wavelengths (400 nm to 480 nm) and had a moderate reflectance value. Moving towards the higher wavelengths (500 nm to 580 nm), P2.0 showed the lowest reflectance value, while P3.0 exhibited the lowest reflectance value at higher wavelengths ranging from 580 nm to 730 nm compared to other formulations. The high visible spectrum reflectance is correlated with the high L* values of bread, which suggests a lighter-colored bread. The lighter color of bread is usually considered more appealing and of superior quality [58].

It is well documented by Udomkun et al. (2022) that bread with high L* and WI values is often associated with low YI and BI values. This is associated with an attractive appearance and improved shelf life of the bread loaves. Further, such color characteristics improve the visual appeal and sensory attributes of bread, resulting in persistent satisfaction and acceptance among consumers [59]. Our investigation revealed similar findings, with P0.5 demonstrating optimal colorimetric properties that may correlate with a longer shelf life of bread and higher acceptance among consumers.

### 3.7. Surface Topology

**Figure 7:**
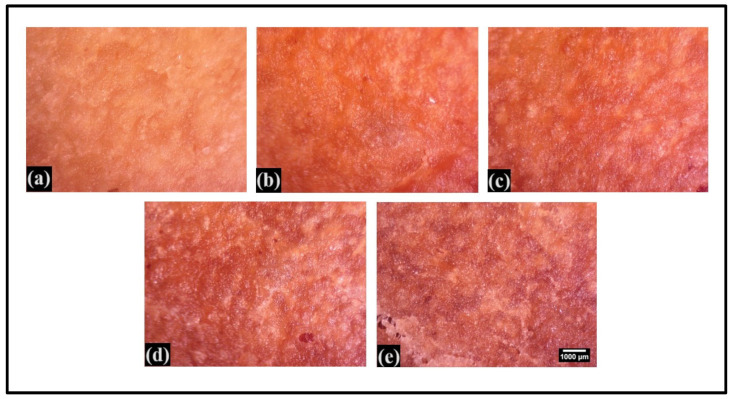
The surface topology of the crust of whole wheat bread (WWB) formulations. WWB formulations containing chitosan lactate (CL) in different concentrations: P0.0 (control, absence of CL), P0.5, P1.0, P2.0, and P3.0 (0.5%, 1.0%, 2.0%, and 3.0% of CL). (**a**) P0.0, (**b**) P0.5, (**c**) P1.0, (**d**) P2.0, (**e**) P3.0. Scale bar: 1000 μm—the same magnification for all images.

The surface characteristics of the bread crust were analyzed using a StereoZoom microscope to determine attributes such as roughness and texture [60]. Analysis of the micrographs suggests that the surface of P0.0 consisted of shiny white structures (Figure 7). These structures can be explained by the presence of caramelized sugar residues on the bread’s surface. The occurrence of these shiny white structures can be accounted for by the Maillard reaction, which has been reported to promote the caramelization of residual sugars [61]. In the CL-containing formulations, there was an increase in the number of shiny white structures with the increase in the CL content. This suggested that an increase in the CL content increased the Maillard reaction [30], a well-documented fact that correlates well with the colorimetric studies reported in the previous section.

**Figure 8:**
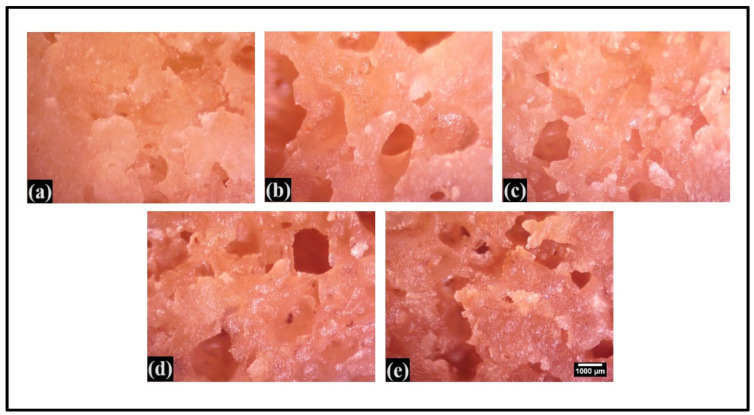
The surface topology of the crumb of whole wheat bread (WWB) formulations. WWB formulations containing chitosan lactate (CL) in different concentrations: P0.0 (control, absence of CL), P0.5, P1.0, P2.0, and P3.0. (**a**) P0.0, (**b**) P0.5, (**c**) P1.0, (**d**) P2.0, (**e**) P3.0 (0.5%, 1.0%, 2.0%, and 3.0% of CL). Scale bar: 1000 μm—the same magnification for all images.

Micrographs of the crumb of WWB formulations offer insight into the porosity of bread. In the case of P0.0, only a few small pores were observed (Figure 8). Larger pores were found in CL-laden formulations (P0.5, P1.0, and P2.0), with P0.5 showing a higher occurrence of larger pores. Moving towards P3.0, it was noticed that the pores were smaller in size. However, it had a uniform distribution. The occurrence of larger pores in P0.5 might have contributed towards enhanced loaf height and, consequently, led to the development of soft and desirable crumbs [33]. A similar pattern of porosity was observed during the visual and physical examination of the bread formulations (Figure 1).

### 3.8. Texture Analysis

#### 3.8.1. Texture Profile Analysis (TPA)

**Figure 9:**
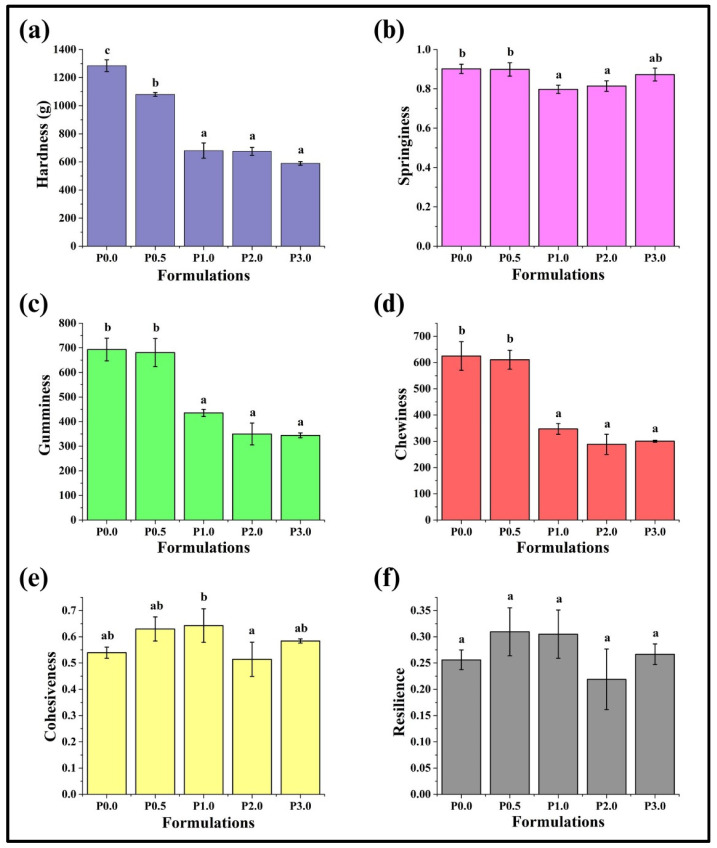
Bar graphs representing texture profile analysis (TPA) of whole wheat bread (WWB) formulations: (**a**) hardness, (**b**) springiness, (**c**) gumminess, (**d**) chewiness, (**e**) cohesiveness, and (**f**) resilience. WWB formulations containing chitosan lactate (CL) in different concentrations: P0.0 (control, absence of CL), P0.5, P1.0, P2.0, and P3.0 (0.5%, 1.0%, 2.0%, and 3.0% of CL). The values denoted in the graphs are the average ± SD of triplicate samples. Bars with different letters differ significantly (*p* < 0.05).

Texture profile analysis (TPA) is essential for both quality assurance and the development of food products. It evaluates properties such as cohesiveness, hardness, springiness, resilience, gumminess, and chewiness. Understanding these characteristics is essential for determining how consumers perceive the quality of bread and improving recipes and manufacturing techniques [62]. The amount of force needed to produce a specific deformation (in our case, 50% of the original height (2 cm)) is often referred to as hardness [63]. It was observed that the average hardness values of WWB formulations ranged from 1284 g to 589 g (Figure 9a). P0.0 showed the highest hardness (1284.52 ± 41.73 g) among all the formulations, followed by a significant reduction in hardness of P0.5 (*p* < 0.05). Further increases in the CL content reduced the hardness values in P1.0, P2.0, and P3.0 compared to P0.5 (*p* < 0.05). However, the changes in the hardness values of P1.0, P2.0, and P3.0 were statistically insignificant (*p* > 0.05). This result can be explained by the interaction of the chitosan derivatives with the gluten proteins present in the bread dough, which can consequently affect the formation of the gluten network. These interactions can introduce alterations in the bread structure, resulting in reduced bread firmness [64].

Springiness is another important characteristic of bread, directly related to its freshness. It indicates the crumbs’ elastic resiliency when a compressive force is released [62]. The average springiness values of WWB samples ranged from 0.90 to 0.79 (Figure 9b). Springiness values of P0.0, P0.5, and P3.0 were comparable (*p* > 0.05). It can be seen that the springiness of P1.0 and P2.0 was lower than the other formulations. The springiness of P1.0, P2.0, and P3.0 presented similar values (*p* > 0.05). Gumminess can be described as the amount of energy needed to transform a semi-solid substance into a swallowable form. On the other hand, chewiness is the duration it takes to chew a sample at a consistent force until it attains a suitable consistency for swallowing [25]. The average values of gumminess and chewiness of WWB samples ranged from 692.98 to 344.06 and 625.05 to 300.07, respectively. The values of gumminess and chewiness of P0.0 and P1.0 were similar (*p* > 0.05), whereas these values were markedly lowered in P1.0, P2.0, and P3.0 compared to P0.0 and P1.0 (*p* < 0.05). Another essential aspect of bread in determining its textural characteristics is cohesiveness. Cohesiveness refers to the extent to which a material is compressed completely between teeth before it ruptures. The average cohesiveness values of WWB formulations ranged from 0.51 to 0.64 (Figure 9e). It was found that the cohesiveness values of all the formulations were statistically similar (*p* > 0.05). Resilience was the last characteristic of TPA analysis, which can be described as the level of resistance a material offers against deformation [65]. The average value of resilience of WWB samples lies between 0.21 and 0.30 (Figure 9f). As illustrated in Figure 9, the resilience values of WWB samples were statistically similar (*p* > 0.05).

#### 3.8.2. Stress Relaxation Profile (SRP)

**Figure 10:**
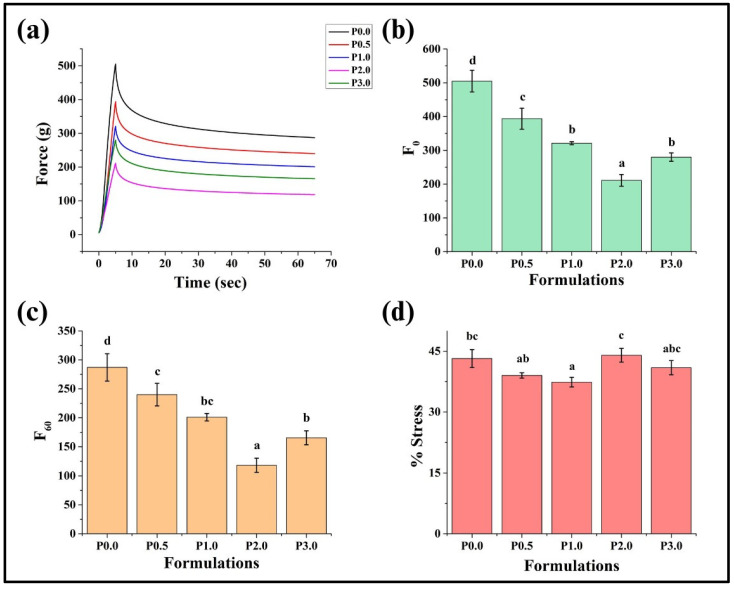
Bar graphs representing: (**a**) stress relaxation profile of whole wheat bread (WWB) formulations and its parameters (**b**) F0, (**c**) F60, and (**d**) % stress. WWB formulations containing chitosan lactate (CL) in different concentrations: P0.0 (control, absence of CL), P0.5, P1.0, P2.0, and P3.0 (0.5%, 1.0%, 2.0%, and 3.0% of CL). The values denoted in the graphs are the average ± SD of triplicate samples. Bars with different letters differ significantly (*p* < 0.05).

The stress relaxation profile is necessary for assessing bread quality as it reveals the viscoelastic attributes of the bread (Figure 10a) [66]. F_0_ is the maximum force achieved in the SR profile, which helped predict the firmness of the WWB formulations. A decreasing trend in F_0_ values was observed from P0.0 to P2.0 (*p* < 0.05), while the F_0_ value of P3.0 was statistically similar to P2.0 (*p* > 0.05) (Figure 10b). The decreasing trend of F_0_ values suggests the reduction in firmness of the WWB formulations, which can be attributed to the interaction between CL and the gluten protein present in the bread dough [64]. While the strained condition was maintained for 60 s, the force values exponentially decreased after F_0_ to reach a basal level (F_60_). The F_60_ values of the prepared WWB formulations exhibited a similar trend as in F_0_ (Figure 10c). Further, the %SR of WWB formulations was calculated using F_0_ and F_60_ values. The %SR values of WWB formulations were lower than 50% and were comparable (*p* > 0.05), suggesting the predominant elastic nature of the WWB formulations (Figure 10d). In summary, the addition of CL did not affect the viscoelasticity of the WWB formulations.

Overall, extremely hard bread is usually considered undesirable as it becomes difficult to consume and is often associated with poor quality and staling. In contrast, excessively soft bread may suggest incomplete development of the gluten network or a high moisture content, which can negatively impact the shelf life of bread. Bread with moderate firmness is usually preferable because it provides a balance between structural integrity and palatability [67]. Further, bread with higher gumminess, chewiness, and springiness values is often associated with enhanced textural properties and prolonged shelf life [68]. In our case, P0.5 fits completely with the above characteristics as it demonstrated moderate firmness and high springiness, gumminess, and chewiness values that correspond to improved textural properties and prolonged shelf life of the bread.

### 3.9. Microbiological Analysis

**Figure 11:**
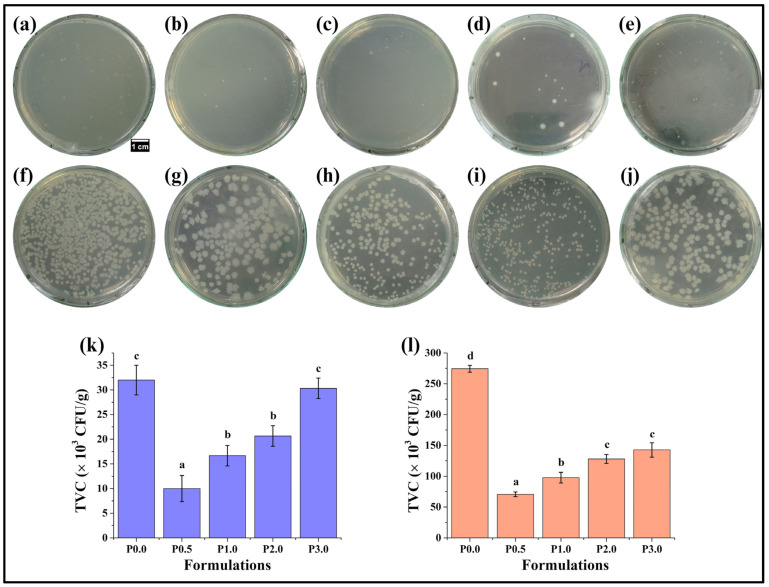
The number of colonies on day 0 of storage of whole wheat bread (WWB): (**a**) P0.0, (**b**) P0.5, (**c**) P1.0, (**d**) P2.0, and (**e**) P3.0. The number of colonies on 2nd day of storage: (**f**) P0.0, (**g**) P0.5, (**h**) P1.0, (**i**) P2.0, and (**j**) P3.0 (diameter of Petri plates = 8.5 cm), and bar graphs representing total plate count (×10^3^ CFU/g) of WWB formulations: (**k**) on day 0 of storage and (**l**) on 2nd day of storage. WWB formulations containing chitosan lactate (CL) in different concentrations: P0.0 (control, absence of CL), P0.5, P1.0, P2.0, and P3.0 (0.5%, 1.0%, 2.0%, and 3.0% of CL). The values denoted in the graphs are the average ± SD of triplicate samples. Bars with different letters differ significantly (*p* < 0.05).

Analysis of microbial growth is an important analysis that can directly indicate the shelf life of the food products and ensure food safety [69]. The microbiological growth analysis of the prepared WWB formulations was carried out for 2 days. On day 0, it was found that the total viable count (TVC) of P0.0 and P3.0 was higher than that of other formulations (*p* < 0.05) (Figure 11k). In contrast, the TVC of P0.5 was the lowest among all the formulations (*p* < 0.05). Furthermore, the TVC of P1.0 and P2.0 was comparable TVC (*p* > 0.05). Further, on the second day of microbiological analysis of the WWB formulations, the TVC of P0.0 was the highest, whereas the lowest TVC was found in P0.5 (*p* < 0.05) (Figure 11l). In P1.0 and P2.0, there was an increase in TVC compared to P0.5 (*p* < 0.05). A subsequent increase in TVC was observed in P3.0; however, the TVC of P3.0 was comparable to P2.0 (*p* > 0.05). The above observations suggest that P0.5 demonstrated optimal antimicrobial activity as it could reduce the proliferation of microbes more significantly than others. Researchers have reported that adding chitosan derivatives to bread can significantly reduce the proliferation of microorganisms during storage, leading to prolonged shelf life and maintaining the bread quality [70]. This might explain the optimal antimicrobial behavior observed in P0.5. Further, a study explained that the higher concentration of chitosan derivatives can form aggregates, which might reduce their ability to interact with the microbes, thereby resulting in lower antimicrobial activity [71]. This statement supports our investigation as the number of colonies gradually increased with the increasing concentration of CL in WWB formulations. Hence, it can be expected that a high concentration of CL in WWB formulations (P1.0, P2.0, and P3.0) might have led to the formation of aggregates. This, in turn, might have resulted in lowering the antimicrobial resistance properties of P1.0, P2.0, and P3.0.

Additionally, it has been previously reported that the maximum permissible limit for total viable count (TVC) in bakery products such as bread, biscuits, and cake should be less than 100,000 CFU/g [72]. In our investigation, we observed that the TVC of P0.0 (control sample without CL) had exceeded the permissible limit on the second day of storage, which indicates the spoilage of the P0.0 formulation (Figure 11l). On the other hand, the TVCs of P0.5 and P1.0 on the second day of storage were less than the maximum permissible limit. Further, in the case of P2.0 and P3.0, the TVC exceeded the maximum permissible limit. Expectedly, despite exceeding the maximum permitted limit, the TVC of P2.0 and P3.0 was less than that of the P0.0 formulation. In other words, the presence of CL led to the reduction in microbial load in the WWB formulations, which is in accordance with the previously reported literature. In conclusion, P0.5 demonstrated optimum antimicrobial activity, which can lead to a prolonged shelf life of WWB [70].

### 3.10. Sensory Analysis

**Figure 12:**
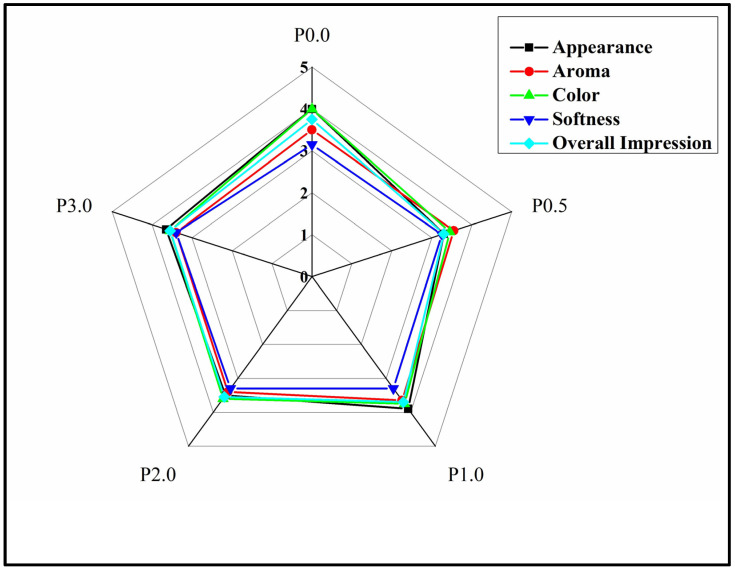
Sensory evaluation of whole wheat bread (WWB) formulations with 5-point hedonic scale denoting scores from 1 to 5 as per consumer perception. WWB formulations containing chitosan lactate (CL) in different concentrations: P0.0 (control, absence of CL), P0.5, P1.0, P2.0, and P3.0.

Table 2 represents the average scores of sensory analysis of the WWB formulations. The sensory parameters that were assessed in this study include appearance, color, aroma, softness, and overall impression of the WWB formulations. It is a well-documented fact that the average scores reflect consumer perceptions of the sensory qualities of the WWB formulations, with higher scores indicating positive responses and lower scores suggesting negative responses. The sensory parameters (appearance, aroma, color, softness, and overall impression) of all the WWB formulations were found to be statistically similar (*p* > 0.05). Hence, it can be assumed that the addition of CL in WWB formulations did not produce any significant change in the sensory attributes of WWBs [64]. Additionally, a 5-point hedonic scale was constructed to offer enhanced comprehension through a diagrammatic representation (Figure 12). It was found that the sensory attributes (appearance, aroma, color, softness, and overall impression) of all the WWB formulations were comparable (*p* > 0.05).

## 4. Conclusions

This study aimed to explore the impact of varying concentrations of CL on the physicochemical properties of WWBs. Visual and physical inspection revealed that P0.5 (0.5% *w*/*w* of CL) exhibited a reduced Maillard reaction and increased loaf volume compared to other formulations. Subsequently, moisture analysis of the WWB formulations indicated that P0.5 had a higher moisture content than P0.0, while further additions of CL did not result in increased moisture levels. P0.5 also showed lower impedance, facilitating better movement of water molecules. The swelling study results showed that P0.5 had higher swelling index values compared to the control (P0.5), and further addition of CL in WWB formulations (P1.0, P2.0, and P3.0) resulted in comparable SI values. Additionally, P0.5 demonstrated optimal colorimetric characteristics, including high L* and WI values, low YI and BI values, and a high reflectance value, which indicate a prolonged shelf life for the WWB. The microscopic examination revealed larger-sized pores in P0.5, which indicate better fermentation of the WWB. FTIR analysis of the WWB formulations revealed that the addition of CL in WWB resulted in improved interaction and bonding of CL with water molecules. Furthermore, P0.5 demonstrated optimal textural and antimicrobial activity among all the formulations. In the sensory analysis, the addition of CL in WWB formulations did not produce any significant changes. Overall, we can conclude that P0.5 has demonstrated all the optimal characteristics that can lead to improved quality and a prolonged shelf life of the WWB.

## Figures and Tables

**Table 1:** Composition of whole wheat bread (WWB) formulations containing chitosan lactate (CL) in different concentrations: P0.0 (control, absence of CL), P0.5 (0.5% *w*/*w* CL), P1.0 (1.0% *w*/*w* CL), P2.0 (2.0% *w*/*w* CL) and P3.0 (3.0% *w*/*w* CL), respectively.

Formulations	Composition (g)						
	WWF	Water	Yeast	Sugar	Salt	CL	Rice Bran Oil
P0.0	220.00	220.00	7.00	29.00	2.00	0.0	22.00
P0.5	218.90	220.00	7.00	29.00	2.00	1.1	22.00
P1.0	217.80	220.00	7.00	29.00	2.00	2.2	22.00
P2.0	215.60	220.00	7.00	29.00	2.00	4.4	22.00
P3.0	213.40	220.00	7.00	29.00	2.00	6.6	22.00

**Table 2:** Average scores of sensory evaluations of whole wheat bread (WWB) formulations. WWB formulations containing chitosan lactate (CL) in different concentrations: P0.0 (control, absence of CL), P0.5, P1.0, P2.0, and P3.0 (0.5%, 1.0%, 2.0%, and 3.0% of CL). The values denoted in the table are the average ± SD of 20 panelists. All the values were statistically insignificant and represented with the letter “a” (*p* > 0.05).

Parameters	P0.0	P0.5	P1.0	P2.0	P3.0
Appearance	4.00 ± 0.70 ^a^	3.30 ± 1.26 ^a^	3.90 ± 0.70 ^a^	3.50 ± 1.02 ^a^	3.65 ± 1.23 ^a^
Aroma	3.50 ± 1.10 ^a^	3.55 ± 0.92 ^a^	3.65 ± 0.79 ^a^	3.40 ± 0.86 ^a^	3.40 ± 0.86 ^a^
Color	4.00 ± 1.09 ^a^	3.45 ± 1.28 ^a^	3.75 ± 0.82 ^a^	3.60 ± 1.15 ^a^	3.55 ± 0.97 ^a^
Softness	3.15 ± 1.23 ^a^	3.25 ± 1.08 ^a^	3.30 ± 1.05 ^a^	3.30 ± 0.90 ^a^	3.30 ± 0.96 ^a^
Overall Impression	3.75 ± 1.04 ^a^	3.30 ± 1.14 ^a^	3.70 ± 0.71 ^a^	3.55 ± 1.07 ^a^	3.55 ± 0.92 ^a^

## Data Availability

The original contributions presented in the study are included in the article, further inquiries can be directed to the corresponding author.

## References

[1] Kweon M., Slade L., Levine H., Gannon D.J. (2014). Cookie-versus cracker-baking—What’s the difference? Flour functionality requirements explored by src and alveography. Crit. Rev. Food Sci. Nutr..

[2] Giannou V., Kessoglou V., Tzia C.J. (2003). Quality and safety characteristics of bread made from frozen dough. Trends Food Sci. Technol..

[3] Pico J., Bernal J., Gómez M.J. (2015). Wheat bread aroma compounds in crumb and crust: A review. Food Res. Int..

[4] Fardet A., Leenhardt F., Lioger D., Scalbert A., Rémésy C.J. (2006). Parameters controlling the glycaemic response to breads. Nutr. Res. Rev..

[5] Juntunen K.S., Niskanen L.K., Liukkonen K.H., Poutanen K.S., Holst J.J., Mykkänen H.M. (2002). Postprandial glucose, insulin, and incretin responses to grain products in healthy subjects. Am. J. Clin. Nutr..

[6] Morris K.L., Zemel M.B. (1999). Glycemic index, cardiovascular disease, and obesity. Nutr. Rev..

[7] Slavin J. (2004). Whole grains and human health. Nutr. Res. Rev..

[8] Liu R.H. (2007). Whole grain phytochemicals and health. J. Cereal Sci..

[9] Doblado-Maldonado A.F., Pike O.A., Sweley J.C., Rose D.J. (2012). Key issues and challenges in whole wheat flour milling and storage. J. Cereal Sci..

[10] Galliard T., Collins A. (1988). Effects of oxidising improvers, an emulsifier, fat and mixer atmosphere on the performance of wholemeal flour in the chorleywood bread process. J. Cereal Sci..

[11] Wang J., Rosell C.M., de Barber C.B. (2002). Effect of the addition of different fibres on wheat dough performance and bread quality. Food Chem..

[12] Ferrero C. (2017). Hydrocolloids in wheat breadmaking: A concise review. Food Hydrocoll..

[13] Gómez M., Ronda F., Caballero P.A., Blanco C.A., Rosell C.M. (2007). Functionality of different hydrocolloids on the quality and shelf-life of yellow layer cakes. Food Hydrocoll..

[14] Tabara A., Miyajima C., Moki N., Kasahara F., Seguchi M. (2016). Improvement of bread making properties by the addition of alginates. Food Sci. Technol. Res..

[15] Zhang D., Lin Z., Lei W., Zhong G. (2020). Synergistic effects of acetylated distarch adipate and sesbania gum on gelatinization and retrogradation of wheat starch. Int. J. Biol. Macromol..

[16] Muzzarelli R.A. (1996). Chitosan-based dietary foods. Carbohydr. Polym..

[17] Shariatinia Z., Jalali A.M. (2018). Chitosan-based hydrogels: Preparation, properties and applications. Int. J. Biol. Macromol..

[18] No H., Meyers S., Prinyawiwatkul W., Xu Z. (2007). Applications of chitosan for improvement of quality and shelf life of foods: A review. J. Food Sci..

[19] Priyadarshi R., Rhim J.-W. (2020). Chitosan-based biodegradable functional films for food packaging applications. Innov. Food Sci. Emerg. Technol..

[20] Rakcejeva T., Rusa K., Dukalska L., Kerch G. (2010). Effect of chitosan and chitooligosaccharide lactate on free lipids and reducing sugars content and on wheat bread firming. Eur. Food Res. Technol..

[21] Sharadanant R., Khan K. (2003). Effect of Hydrophilic Gums on the Quality of Frozen Dough: II. Bread Characteristics. Cereal Chem..

[22] Qureshi D., Pattanaik S., Mohanty B., Anis A., Kulikouskaya V., Hileuskaya K., Agabekov V., Sarkar P., Maji S., Pal K. (2022). Preparation of novel poly (vinyl alcohol)/chitosan lactate-based phase-separated composite films for UV-shielding and drug delivery applications. Polym. Bull..

[23] Nguyen T.T.B., Hein S., Ng C., Stevens W.F. (2007). Molecular stability of chitosan in acid solutions stored at various conditions. J. Appl. Polym. Sci..

[24] Ahmad S., Naz A., Usman M., Amjad A., Pasha I., Farooq U. (2021). Impediment effect of chemical agents (additives) on gluten development in cookie dough. J. Food Sci. Technol..

[25] Alam S.S., Bharti D., Pradhan B.K., Sahu D., Dhal S., Kim N.M., Jarzębski M., Pal K. (2022). Analysis of the Physical and Structure Characteristics of Reformulated Pizza Bread. Foods.

[26] Dhal S., Anis A., Shaikh H.M., Alhamidi A., Pal K. (2023). Effect of Mixing Time on Properties of Whole Wheat Flour-Based Cookie Doughs and Cookies. Foods.

[27] Jain A., Pradhan B.K., Mahapatra P., Ray S.S., Chakravarty S., Pal K. (2020). Development of a low-cost food color monitoring system. Color Res. Appl..

[28] Pathak D., Majumdar J., Raychaudhuri U., Chakraborty R. (2016). Characterization of physicochemical properties in whole wheat bread after incorporation of ripe mango peel. J. Food Meas. Charact..

[29] Humphries J.M., Graham R.D., Mares D.J. (2004). Application of reflectance colour measurement to the estimation of carotene and lutein content in wheat and triticale. J. Cereal Sci..

[30] Silva H.A., Paiva E.G., Lisboa H.M., Duarte E., Cavalcanti-Mata M., Gusmao T., de Gusmao R. (2020). Role of chitosan and transglutaminase on the elaboration of gluten-free bread. J. Food Sci. Technol..

[31] Kosaraju S.L., Weerakkody R., Augustin M.A. (2010). Chitosan− glucose conjugates: Influence of extent of Maillard reaction on antioxidant properties. J. Agric. Food Chem..

[32] Besbes E., Jury V., Monteau J.-Y., Le Bail A. (2013). Characterizing the cellular structure of bread crumb and crust as affected by heating rate using X-ray microtomography. J. Food Eng..

[33] Munteanu G.-M., Voicu G., Ferdeş M., Ştefan E.-M., Constantin G.-A., Tudor P. (2019). Dynamics of fermentation process of bread dough prepared with different types of yeast. Sci. Study Res. Chem. Chem. Eng. Biotechnol. Food Ind..

[34] Dou X., Hao Y., Sun Y., Yang P., Liu L., He Y., Shi Y., Yang C., Chen F. (2024). A novel baking additive: Preparation, characterization, and application of chitosan hydrochloride/carboxymethyl starch sodium nano-gel for wheat bread. Food Hydrocoll..

[35] Dabija A., Codină G.G., Fradinho P. (2017). Effect of yellow pea flour addition on wheat flour dough and bread quality. Rom. Biotechnol. Lett..

[36] Lafarga T., Gallagher E., Walsh D., Valverde J., Hayes M. (2013). Chitosan-containing bread made using marine shellfishery byproducts: Functional, bioactive, and quality assessment of the end product. J. Agric. Food Chem..

[37] Mollakhalili-Meybodi N., Sheidaei Z., Khorshidian N., Nematollahi A., Khanniri E. (2022). Sensory attributes of wheat bread: A review of influential factors. J. Food Meas. Charact..

[38] Shaw G.S., Pandey P.M., Yogalakshmi Y., Banerjee I., Al-Zahrani S.M., Anis A., Pal K. (2017). Synthesis and Assessment of Novel Gelatin–Chitosan Lactate Cohydrogels for Controlled Delivery and Tissue Engineering Applications. Polym. Technol. Eng..

[39] Kertész Á., Hlaváčová Z., Vozáry E., Staroňová L. (2015). Relationship between moisture content and electrical impedance of carrot slices during drying. Int. Agrophys..

[40] Wessels R., Wentzel B., Labuschagne M. (2020). Solvent retention capacity and swelling index of glutenin in hard red wheat flour as possible indicators of rheological and baking quality characteristics. J. Cereal Sci..

[41] Kowalczyk D., Karaś M., Kordowska-Wiater M., Skrzypek T., Kazimierczak W. (2023). Inherently acidic films based on chitosan lactate-doped starches and pullulan as carries of nisin: A comparative study of controlled-release and antimicrobial properties. Food Chem..

[42] Kanazawa S., Sanabria M., Monteiro M. (2021). Influence of the fermentation methods on the resistant starch formation by X-ray diffraction. SN Appl. Sci..

[43] Sadat A., Joye I.J. (2020). Peak fitting applied to fourier transform infrared and raman spectroscopic analysis of proteins. Appl. Sci..

[44] Tyagi P., Chauhan A.K. (2020). Aparna Optimization and characterization of functional cookies with addition of Tinospora cordifolia as a source of bioactive phenolic antioxidants. LWT.

[45] Walrafen G.E., Hokmabadi M.S., Yang W.-H. (1986). Raman isosbestic points from liquid water. J. Chem. Phys..

[46] De Ninno A., De Francesco M. (2018). ATR-FTIR study of the isosbestic point in water solution of electrolytes. Chem. Phys..

[47] Garcia-Valle D.E., Bello-Pérez L.A., Agama-Acevedo E., Alvarez-Ramirez J. (2021). Effects of mixing, sheeting, and cooking on the starch, protein, and water structures of durum wheat semolina and chickpea flour pasta. Food Chem..

[48] Pulatsu E., Su J.-W., Kenderes S.M., Lin J., Vardhanabhuti B., Lin M. (2021). Effects of ingredients and pre-heating on the printing quality and dimensional stability in 3D printing of cookie dough. J. Food Eng..

[49] Yang S., Dhital S., Zhang M.-N., Wang J., Chen Z.-G. (2022). Structural, gelatinization, and rheological properties of heat-moisture treated potato starch with added salt and its application in potato starch noodles. Food Hydrocoll..

[50] Yang S., Zhang M.-N., Shan C.-S., Chen Z.-G. (2021). Evaluation of cooking performance, structural properties, storage stability and shelf life prediction of high-moisture wet starch noodles. Food Chem..

[51] Kerch G., Zicans J., Meri R.M. (2010). The effect of chitosan oligosaccharides on bread staling. J. Cereal Sci..

[52] Tolve R., Simonato B., Rainero G., Bianchi F., Rizzi C., Cervini M., Giuberti G. (2021). Wheat bread fortification by grape pomace powder: Nutritional, technological, antioxidant, and sensory properties. Foods.

[53] Sahu D., Bharti D., Kim D., Sarkar P., Pal K. (2021). Variations in microstructural and physicochemical properties of candelilla wax/rice bran oil–derived oleogels using sunflower lecithin and soya lecithin. Gels.

[54] Michalska A., Amigo-Benavent M., Zielinski H., del Castillo M.D. (2008). Effect of bread making on formation of Maillard reaction products contributing to the overall antioxidant activity of rye bread. J. Cereal Sci..

[55] Ndlala F.N., Onipe O.O., Mokhele T.M., Anyasi T.A., Jideani A.I.O. (2019). Effect of wheat bran incorporation on the physical and sensory properties of a south african cereal fried dough. Foods.

[56] Jung H., Sato T. (2013). Comparison between the color properties of whiteness index and yellowness index on the CIELAB. J. Korean Dye. Process. Soc..

[57] Popov-Raljić J.V., Mastilović J.S., Laličić-Petronijević J.G., Popov V.S. (2009). Investigations of bread production with postponed staling applying instrumental measurements of bread crumb color. Sensors.

[58] Guiotto E.N., Tomás M.C., Haros C.M. (2020). Development of highly nutritional breads with by-products of chia (*Salvia hispanica* L.) seeds. Foods.

[59] Udomkun P., Masso C., Swennen R., Romuli S., Innawong B., Kuate A.F., Akin-Idowu P.E., Alakonya A., Vanlauwe B. (2022). Comparative study of physicochemical, nutritional, phytochemical, and sensory properties of bread with plantain and soy flours partly replacing wheat flour. Food Sci. Nutr..

[60] Onipe O.O., Beswa D., Jideani A.I.O. (2020). Confocal laser scanning microscopy and image analysis for elucidating crumb and crust microstructure of bran-enriched south african fried dough and batter. Foods.

[61] Helou C., Jacolot P., Niquet-Léridon C., Gadonna-Widehem P., Tessier F.J. (2016). Maillard reaction products in bread: A novel semi-quantitative method for evaluating melanoidins in bread. Food Chem..

[62] Tóth M., Kaszab T., Meretei A. (2022). Texture profile analysis and sensory evaluation of commercially available gluten-free bread samples. Eur. Food Res. Technol..

[63] Peleg M. (2019). The instrumental texture profile analysis revisited. J. Texture Stud..

[64] Pyo S.-H., Moon C.-R., Park S.-W., Choi J.-Y., Park J.-D., Sung J.M., Choi E.-J., Son Y.-J. (2024). Quality and staling characteristics of white bread fortified with lysozyme-hydrolyzed mealworm powder (*Tenebrio molitor* L.). Curr. Res. Food Sci..

[65] Yildiz Ö., Yurt B., Baştürk A., Toker Ö.S., Yilmaz M.T., Karaman S., Dağlıoğlu O. (2013). Pasting properties, texture profile and stress–relaxation behavior of wheat starch/dietary fiber systems. Food Res. Int..

[66] Figueroa J.D.C., Hernández Z.J.E., Rayas-Duarte P., Peña R.J. (2013). Stress relaxation and creep recovery tests performed on wheat kernels versus doughs: Influence of glutenins on rheological and quality properties. Cereal Foods World.

[67] Bhise S., Kaur A. (2014). Baking quality, sensory properties and shelf life of bread with polyols. J. Food Sci. Technol..

[68] Wanjuu C., Abong G., Mbogo D., Heck S., Low J., Muzhingi T. (2018). The physiochemical properties and shelf-life of orange-fleshed sweet potato puree composite bread. Food Sci. Nutr..

[69] Tarlak F. (2023). The Use of Predictive Microbiology for the Prediction of the Shelf Life of Food Products. Foods.

[70] Ma Z., Garrido-Maestu A., Jeong K.C. (2017). Application, mode of action, and in vivo activity of chitosan and its micro-and nanoparticles as antimicrobial agents: A review. Carbohydr. Polym..

[71] Li J., Zhuang S. (2020). Antibacterial activity of chitosan and its derivatives and their interaction mechanism with bacteria: Current state and perspectives. Eur. Polym. J..

[72] Ali M.A., Hashish M.H., Fekry M.M. (2023). Microbiological quality of some packed and unpacked bread products in Alexandria, Egypt. J. Egypt. Public Health Assoc..

